# A Neural Mechanism for Time-Window Separation Resolves Ambiguity of Adaptive Coding

**DOI:** 10.1371/journal.pbio.1002096

**Published:** 2015-03-11

**Authors:** K. Jannis Hildebrandt, Bernhard Ronacher, R. Matthias Hennig, Jan Benda

**Affiliations:** 1 Cluster of Excellence "Hearing4all", Department for Neuroscience, University of Oldenburg, Oldenburg, Germany; 2 Department of Biology, Humboldt-Universität zu Berlin, Berlin, Germany; 3 Institute for Neurobiology, Eberhard Karls Universität Tübingen, Tübingen, Germany; University of Minnesota, UNITED STATES

## Abstract

The senses of animals are confronted with changing environments and different contexts. Neural adaptation is one important tool to adjust sensitivity to varying intensity ranges. For instance, in a quiet night outdoors, our hearing is more sensitive than when we are confronted with the plurality of sounds in a large city during the day. However, adaptation also removes available information on absolute sound levels and may thus cause ambiguity. Experimental data on the trade-off between benefits and loss through adaptation is scarce and very few mechanisms have been proposed to resolve it. We present an example where adaptation is beneficial for one task—namely, the reliable encoding of the pattern of an acoustic signal—but detrimental for another—the localization of the same acoustic stimulus. With a combination of neurophysiological data, modeling, and behavioral tests, we show that adaptation in the periphery of the auditory pathway of grasshoppers enables intensity-invariant coding of amplitude modulations, but at the same time, degrades information available for sound localization. We demonstrate how focusing the response of localization neurons to the onset of relevant signals separates processing of localization and pattern information temporally. In this way, the ambiguity of adaptive coding can be circumvented and both absolute and relative levels can be processed using the same set of peripheral neurons.

## Abstract 2

Neuronal data, computational modeling, and behavioral experiments reveal how the conflict between sensory adaptation and sound localization is resolved in the grasshopper auditory system, allowing processing of both absolute and relative sound levels.

## Author Summary

Smell, vision, hearing—virtually all of our senses adapt their sensitivity to cope with the varying environment. Adaptation removes information about absolute stimulus intensity available to the brain, as this information is usually of little relevance for sensory representation. For some tasks, however, knowledge of absolute stimulus intensities is essential. How sensory pathways cope with this conflict remains an open question. We addressed this question in the grasshopper auditory system, in which comparison of absolute intensities of conspecific calls at both ears is crucial for mate localization. We recorded activity from three levels in the auditory pathway, showing that adaptation in the peripheral auditory system indeed removes information about absolute intensities. We discovered that strong negative feedback restricts coding of sound direction in the central auditory system to the very beginning of a stimulus, when peripheral adaptation has not yet acted. By using a computational model, we show that this central mechanism enables localization of the sound source over a wide range of stimulus intensities and that its time course is well matched to the time course of peripheral adaptation. In a final step, we confirmed predictions from our model in behavioral experiments on sound localization.

## Introduction

In many sensory pathways, adaptation serves to adjust neural encoding to the current statistics of the environment and thus enables reliable and invariant representation of relevant aspects within seconds [1–3]. In order to achieve this, response normalization has been shown to remove the signal mean [1,4,5], variance [6–8], or even higher statistical moments from the representation [9] along the sensory pathway. From another point of view, this process could be seen as a filter of some statistical moments of the stimulus. However, although filtering out certain aspects of the stimulus may be desirable for some tasks, it could create ambiguity for others [10,11]. For example, a major function of adaptation is to keep the representation of the stimulus within the dynamic range of the sensory pathway. Hence, adaptation to the mean intensity usually takes place early on in the periphery, often even within receptor cells or after the first synapse [4,12,13]. Consequently, information about the mean intensity should be lost to all later stages of a divergent pathway. How sensory pathways deal with this problem is still under research. It has been suggested that multiplexing the information in different aspects of the response statistics may solve the problem [10], but the identification of mechanisms to read out such a code has been difficult [14]. When possible, adaptation can be placed after divergence of the processing of different aspects [15,16] or more generally spread across larger populations [17,18], but because of limited dynamic range or processing capacity, in many cases this is not a viable solution.

The conflict is particularly prominent in the auditory system, in which differences in the mean sound pressure level between the two ears are used to localize a sound in the horizontal plane [19], while a level-invariant representation of the amplitude modulations of the sound is an important prerequisite for recognition of sound identity [20]. Across a wide range of species, adaptation creates level-invariant representations of amplitude modulations [4,5,7,13], most likely to accommodate for the large range of mean intensities at which even the same sound can be encountered. However, if level-invariance is created by adaptation already in the periphery, central neurons are not able to evaluate inter-aural level differences (ILDs), since mean intensity is already removed from the neural responses.

The auditory system of the grasshopper is perfectly suited to study this potential conflict between peripheral adaptation and central processing of ILDs, since only two features are of major behavioral importance in this system: (1) temporal pattern of the signal that serves both males and females to detect and identify a potential mate [21] and (2) the ILD, on which the animals rely to localize the sound source in order to approach the potential mate [22].

**Fig 1:**
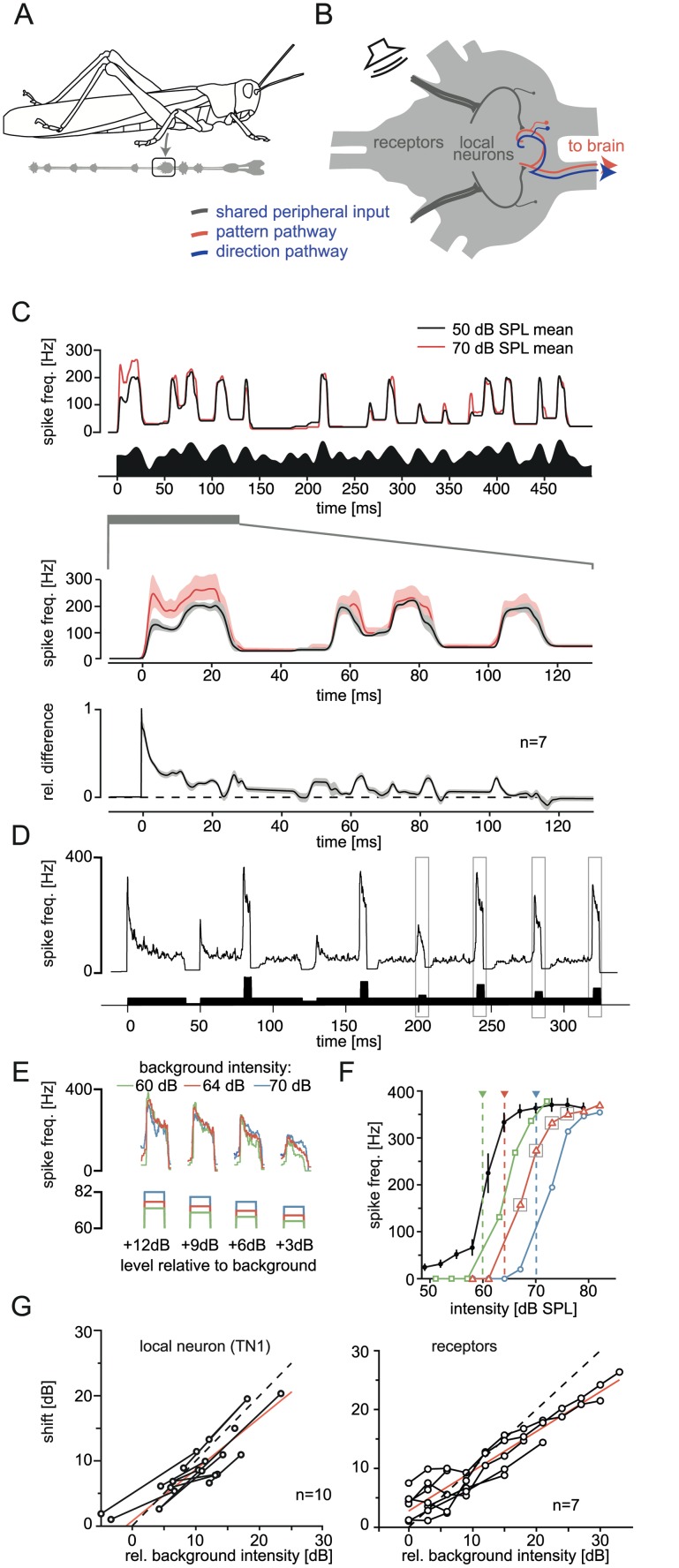
Emergence of level invariance along the pathway. A: In grasshoppers, the ears are located on each side of the first abdominal segment. The first step of neural processing takes place in the metathoracic ganglion. B: Metathoracic auditory pathway of grasshoppers: sound is transduced in receptor neurons in the ear and information is carried to the metathoracic ganglion, where receptors synapse on local neurons (both gray). These connect to ascending neurons, which in turn transfer auditory information about sound pattern (red) and direction (blue) separately up to the brain. C: Mean response of six local neurons (TN1) to an amplitude-modulated (AM) sound (bottom panel). The upper panel shows the spike frequency elicited by the sound played back at two different levels (black and red). The middle panel shows the first 130 ms of the response in more detail; the shaded areas depict standard deviation. The lower panel is the average difference between the responses in the middle panel ([response to louder sound minus response to softer sound]/response to louder sound). D: Example of a measurement of a level-response curve adapted to a given background level in a TN1 neuron. Gray boxes indicate the examples in panels E and F. E: Onset responses to level steps relative to a background level (numbers at the bottom) are independent of different background levels (indicated by line color); the neurons were adapted to a mean level as shown in D. F: Adaptation of level-response curves of a TN1 neuron to different background levels of sound. The black line is the response curve when tested in silence (error bars: standard error of the mean [SEM]); the colored curves represent adapted response curves of the same cell after adaptation to different background levels (dotted vertical lines). G: Shift of the parameterized level-response curves as a function of the background sound for all recorded TN1 (left panel) and receptor cells (right panel). The dashed black line is the prediction of complete compensation of the background level by adaptation, the red line a fit of a straight line to the data. See S1 Data for data underlying panels C–F.

The ears of grasshoppers are located laterally in the first abdominal segment (Fig. 1A). Receptor neurons transduce sound and encode information about the stimulus pattern in action potential frequency [23,24]. The receptor axons enter the metathoracic ganglion, where they synapse on a population of local neurons (Fig. 1B, [25]). In this ganglion, information about the pattern and the directionality of the sound is separated into two channels, represented by different ascending neurons [21,26]. Both channels make use of the same peripheral input from both ears combined in central, ascending neurons. Receptors do not synapse directly onto ascending neurons, but on local neurons only (Fig. 1B). In the case of pattern coding, summation over the peripheral responses from both sides increases the signal-to-noise ratio but leads to a loss of directional information. For directionality, the system evaluates ILD, whereas inter-aural time differences are much too small to be evaluated in grasshoppers (differences of 5–6 μs at most; [27]). In order to evaluate ILDs, the grasshopper ear works as a pressure gradient receiver [28]. In addition, the differences between the peripheral inputs from the two ears are enhanced by contralateral inhibition, emphasizing the directional tuning. The ascending neuron AN2 in locusts is thought to code for the direction of the stimulus [25].

We have previously quantified adaptation at different stages of the metathoracic network and discovered that adaptation takes place at all levels of the pathway [29]. Here we explore how peripheral adaptation influences the central representation of pattern and ILDs and how the network deals with potentially conflicting requirements on adaptation for pattern and ILD coding. In a first step, we experimentally explored at which stage in the metathoracic network of locusts (*Locusta migratoria*) level invariant coding is achieved. Since we had previously observed strong negative feedback in a central direction coding neuron [29], we next tested whether this central mechanism solves the conflict of adaptation on pattern coding and ILD representation. Finally, we used a model based on experimental data from all three stages of the pathway to generate qualitative predictions for behavioral experiments and tested these on male grasshoppers of the species *Chorthippus biguttulus*.

## Results

### Emergence of Level Invariant Coding within the Auditory Pathway

In grasshoppers the peripheral auditory system is located in the metathoracic ganglion (Fig. 1A,B) and exhibits three layers of neurons. Sixty to eighty receptor neurons in the ear encode the sound envelope by action potentials [24]. Receptor neurons form synapses with local interneurons, which pass information onto ascending neurons (Fig. 1B). We hypothesized that neural adaptation should take place before central integration of both sides in ascending neurons in order to be beneficial for pattern coding. However, since local neurons provide the input to ascending neurons of both the pattern and the direction-coding pathway (Fig. 1B), such peripheral adaptation could be detrimental for coding of the direction of a stimulus. In an initial series of experiments we characterized the strength and effect of adaptation at the first two levels of the peripheral system of grasshoppers: receptors and local interneurons.

We first tested whether the firing rate of local interneurons in response to an ongoing, amplitude-modulated (AM) stimulus is independent of the mean intensity of the stimulus. We recorded intracellularly from a local interneuron (TN1), while presenting the same Gaussian white-noise AM stimuli (cutoff 100 Hz) at different mean levels. The upper panel of Fig. 1C shows two responses elicited by such a stimulus presented at two different mean levels. Except for the initially different responses during the first 50 ms (middle panel in Fig. 1C) the firing rate of TN1 was very similar, although both stimuli differed by 20 dB in mean level. We also compared the responses of six different recordings of TN1s to AM stimuli pairwise for the two mean levels over time. The normalized difference between the responses on average dropped to below 15% after 30 ms and then remained low for the entire presentation (Fig. 1C, bottom panel). This demonstrated that at the first steps of processing in the auditory pathway of grasshoppers adaptation to mean sound level enabled intensity-invariant responses already at the input to both the pattern and localization pathway.

In order to quantify level invariance in local interneurons more thoroughly, we tested level-response curves either in silence or during presentation of an adapting background stimulus at different mean intensities (Fig. 1D). If adaptation really results in level invariance, a change of the background level is expected to produce a compensatory shift of the level-response [30]. Indeed, local neurons responded to the level of the test pulses relative to the background instead of responding to the absolute intensity (Fig. 1E). Fig. 1F shows an example of a TN1 that shifts its response curve along the intensity axis for different background levels (dotted vertical lines). A full compensation of the change in mean level by adaptation would correspond to a complete level invariance of responses, indicated by points along the identity line in the left panel of Fig. 1G (slope of 1). The shift of the response curves of TN1 was highly correlated with the background levels for the entire range tested (r = 0.95, *p* < 0.0001), and the slope of 0.83 ± 0.18 (95% confidence interval) indicated that adaptation compensated for most of the change in signal mean (Fig. 1G, left panel). Thus, the TN1 exhibited almost complete adaptation to the background. In order to test how much of this level invariance is inherited from receptor neurons, we performed the same set of experiments while recording intracellularly from seven receptor neurons. Although response curves of receptors were shifted after the presentation of different background levels, the population data of all receptors (Fig. 1G right panel) did reveal less compensation of the presented background intensity by adaptation than local neurons. The slope of the relationship between the intensity of the adapting background and the shift of the response was 0.67 ± 0.08 (linear fit, ± 95% confidence interval; correlation: r = 0.94, *p* < 0.0001), and thus below a slope of one that would be expected for complete level invariance (dashed line, Fig. 1G right). In summary, nearly complete level invariance is already achieved after the first synapse of the grasshopper auditory pathway, thus removing most of the available information about absolute level at each side of the animal before the separation of channels for parallel processing.

### Intrinsic Adaptation of the Central Direction Coding Neuron Is a Potential Mechanism to Enhance Directional Coding

The observed intensity invariant representation of amplitude modulations is likely beneficial for the recognition of song envelopes whose absolute and mean amplitudes depend on the distance between sender and receiver. However, the auditory periphery does not only feed into the pattern processing circuits, but is also used to determine the direction of a sound source (Fig. 1B), for which grasshoppers mainly depend on ILDs [31]. Removing available information about absolute level on each side separately also removes information about ILDs. We next asked how the auditory system of the grasshopper solves this conflict. Since adaptation evolves over time, one potential algorithm would be to restrict reading of ILD information to the stimulus onset.

**Fig 2:**
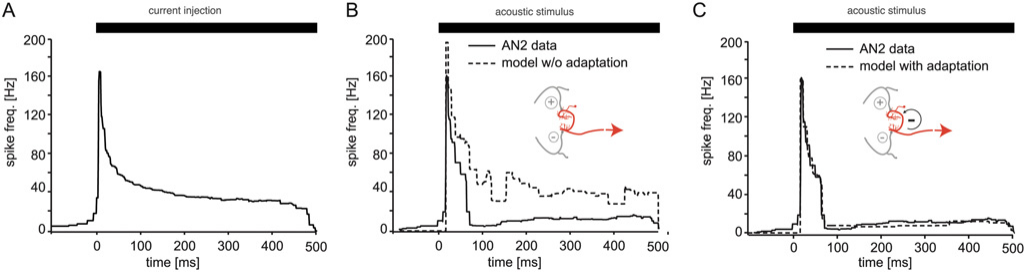
Intrinsic adaptation of the central ILD coding neuron. A: Response adaptation of an AN2 neuron to 500 ms current injection. B: The same neuron stimulated with a 500 ms sound stimulus of constant intensity level (56 dB SPL). The dotted line depicts responses of a model of AN2 that does not include the intrinsic adaptation seen in (A) but receives adapting inputs from the periphery (inset). C: Incorporation of an intrinsic adaptation current into the AN2 model reproduces the strong adaptation in response to acoustic stimuli. See S2 Data for experimental data underlying panels A–C and S1 Code for the code used to generate the modeling results in B and C. w/o: without.

In the grasshopper, direction is encoded in two pairs of ascending interneurons that each receives excitatory input from one side and inhibitory input from the other. In a previous study, we had observed a strong intrinsic activity-dependent adaptation current in one of these neurons (AN2) [29]. When current was injected into the AN2, spike frequency quickly dropped down to very low levels (Fig. 2A) and often completely disappeared at higher current levels. In response to sound stimuli the AN2 displayed an even stronger reduction in firing (Fig. 2B). Since an intrinsic adaptation mechanism restricts firing mostly to the onset of the stimulus that still contains information on absolute sound levels, we hypothesized that intrinsic adaptation could enable the coding of direction despite the observed peripheral adaptation.

### Intrinsic Adaptation Currents Improve Directional Coding

To test our hypothesis and to investigate the effect of central adaptation in ascending neurons on direction coding, we simulated the direction-coding pathway of the grasshopper in a network model. The network consisted of an ipsi- and a contra-lateral population of peripheral neurons that project onto two central, integrating neurons simulated by exponential integrate-and-fire neurons. The peripheral populations were fitted to match the response curves in the experimental data and the observed dynamics of peripheral adaptation [29]. One of the two central, direction-coding neurons was excited by sound from the left and inhibited by sound from the right, the other, vice versa. Parameters for the central neurons were fitted to match the observed spiking responses of AN2 when stimulated with current stimuli. In order to test for the consequences of intrinsic adaptation currents in the central neuron, we ran the model in two versions: with and without an adaptation term in the central neuron (Fig. 2B,C).

The model version without central adaptation failed to reproduce the experimentally observed responses to acoustic stimuli (Fig. 2B). Due to peripheral adaptation, the model showed a marked decrease in spike rate but also displayed a sustained response well above zero. Adding an adaptation current to the central neurons of the model that reproduced the experimental current-injection data resulted in a good match of the firing-rate response of the model to acoustic stimulation with the experimental data (Fig. 2C).

**Fig 3:**
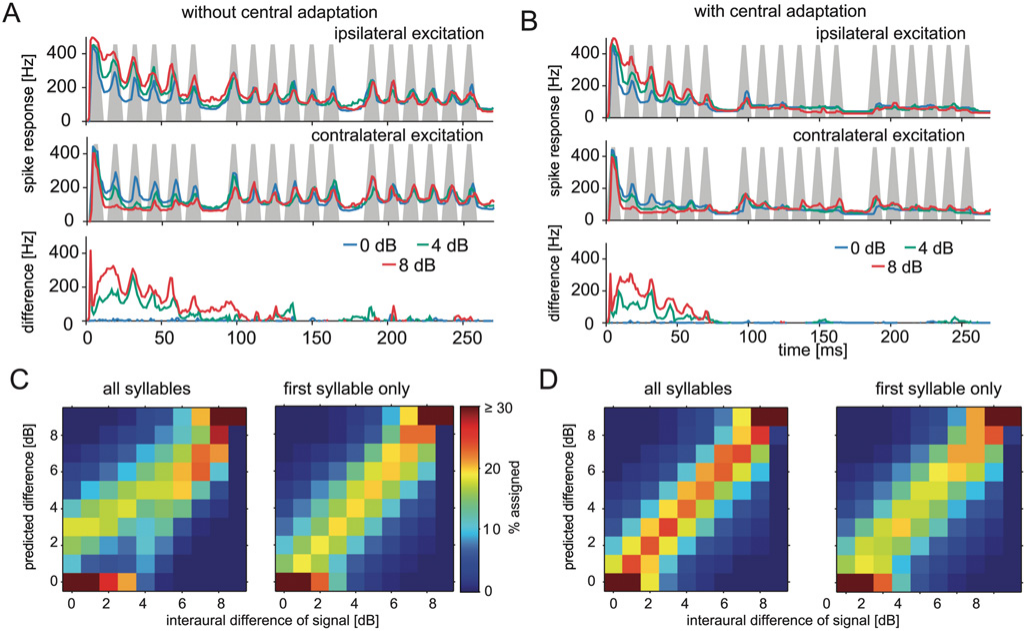
ILD coding improves with additional intrinsic adaptation in the central, ascending neurons. A: Response of a pair of modeled direction coding central neurons to an artificial grasshopper song played back from three directions (indicated by line colors) and the difference between the responses of the ipsi- and contralateral ascending neuron (bottom panel). B: Same simulation, but with intrinsic adaptation added to the model of the ascending/central neurons. C: Decoding of the ILD from the responses as pictured in (A). Responses of the AN pair to combinations of ten directions (ILDs) and 33 mean levels were used and classified for decoding performance of ILDs. Left panel: all syllables of the song taken into account, right panel: only responses to the first syllable of the song were used for classification. D: Classification as in C but with adaptation added to the central neurons in the model. See S2 Code for the code ran to model the network responses and classification of these responses.

We next tested the performance of our two model versions for the encoding of ILD. Responses of the two model neurons to an artificial grasshopper song elicited by presentation of three direction-dependent level differences are presented in Fig. 3A. Initially, for the first sound pulses, the ipsi-laterally excited neuron (upper trace) responded with a higher rate than the contra-laterally excited neuron (middle trace). This was in accordance with the stimulus being louder ipsi-laterally and softer contra-laterally, and this difference in firing rate of the two central neurons potentially encoded the direction of the sound source. However, as the periphery adapted with time and generated a level invariant envelope representation, the responses of the two neurons become very similar to each other and invariant to the direction (Fig. 3A, lower panel). The addition of a central adaptation current did not influence the responses to the initial sound pulses of the artificial song in the model (Fig. 3B). However, for the following sound pulses, the additional central adaptation current strongly reduced the activity in the model AN2 and abolished further representation of the song pattern (Fig. 3B).

Based on the single level example shown in Fig. 3A and Fig. 3B, the grasshopper could discriminate between different directions mostly by a comparison of the onset responses of the two neurons. However, such discrimination needs to be reliable for a much larger range of different sound levels. We therefore tested the discrimination performance of our model with 33 different sound levels and ten sound directions resulting in different ILDs. We then tried to determine the direction of the sound source from the difference of the spiking response of the two model neurons averaged over the entire stimulus. In the model version without central adaptation the direction of sound was reliably detected only for ILDs larger than 6 dB (Fig. 3C, left panel). However, the classification success deteriorated at lower ILDs, for which songs often were either classified as coming from the front (ILD = 0) or from a position further to the side of the animal compared to the original ILD (Fig. 3C left). Therefore, the output of the network did not reliably predict the actual direction if the entire song was taken into account. Only 58.3% of responses were classified within ±1 dB of the original ILD and the mutual information in the confusion matrix in Fig. 3C left panel is 1.03 bits (maximal possible: 3.32). However, more accurate information about sound direction is available by taking only responses to the first syllable into account: performance was much better in this case (Fig. 3C right panel). These modeling results demonstrate again that adaptation to the mean sound level at the very periphery potentially poses a problem for the localization of a sound source despite the initially unadapted responses that convey information about sound direction. Since the peripheral pathway of the grasshopper does create level invariance rapidly within about 50 ms (Fig. 1C and D), the question arises how the animals nevertheless successfully locate the songs of potential mates [22].

When we tested the ability to predict song direction with the second model version (Fig. 3B), the additional, dynamic adaptation current enabled a high ability to discriminate between sound directions (Fig. 3D). Now, the performance of classification becomes more independent of sound level and thus more invariant for overall levels of sound. Both correct assignments and mutual information in the classification matrix (Fig. 3D) increased by about 32% (correct assignments within ILD ± 1 dB: 76.7% versus 58.3%, mutual information: 1.36 versus 1.03 bit). The central adaptation current suppressed the response to the later sound pulses that do not carry directionality information. Therefore, the response to the first sound pulses dominated the overall prediction of the model, offering a solution for the conflict posed by peripheral adaptation. The right panels in Fig. 3C and Fig. 3D demonstrate that this gain was not achieved simply by restricting responses to the first syllable because discrimination performance was better when the modeled response was integrated over the entire song than when only the first syllable was taken into account (76.7% correct within ILD ± 1 dB versus 65.1% correct, 1.36 bit versus 1.15). Thus, the adaptation current in AN2 not only cuts off responses when they become uninformative. The time course of the adaptation enables weighing of responses over time according to how informative they are.

### Dynamics of Central Mechanism Are Matched to Decay of Information in Periphery

**Fig 4:**
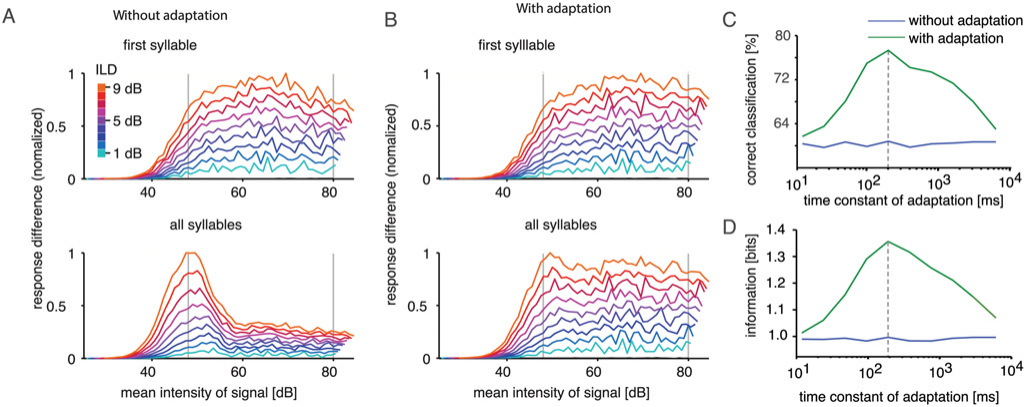
Level invariance of ILD coding and relevance of time course of adaptation. A: Response differences of the model central neuron pair for nine different ILDs played back at different mean levels. Each colored line depicts a single value of ILDs, ranging from 1 dB (light blue) to 9 dB (orange). Upper panel: only responses to first syllable taken into account. Lower panel: responses averaged over the duration of the entire song. Response differences were normalized to the largest response. B: Equivalent to (A), but with added adaptation current in the central neurons. In particular, when averaging over the complete response, ILDs are encoded invariantly with respect to the mean sound intensity (horizontal course of the curves). C: Dependence of classification success on different values of the adaptation time constant of the intrinsic adaptation current in the model. The dashed line indicates the value that best fitted the experimentally observed time course of intrinsic adaptation in AN2 as obtained by current injection (see Fig. 2A). Percentage correct includes correct classification within ±1 dB of the presented ILD. D: Information content of the confusion matrices in dependence on the adaptation time constant. See S2 Code for the code used to model the network responses and classification of these responses.

To investigate the effect of taking the weighted difference of responses rather than simply cutting off AN2 responses, we plotted the response difference between ipsi- and contralateral AN2 model neurons at different mean intensities and ILDs (Fig. 4A and Fig. 4B). Reliable coding of direction would correspond to invariant responses independent of mean sound levels. For both model variants, however, responses to different ILDs depended on the mean level when only responses to the first syllable were taken into account. Response differences were highest at intermediate levels and fell off slightly at lower and higher intensities. Since the classification matrices in Fig. 3C and Fig. 3D were computed for the whole range of mean levels, this dependence on the mean level hindered correct classification of direction. Taking the entire song into account only worsened the classification performance of the model without central adaptation because response differences at the same ILD became even more level dependent (Fig. 4A, lower panel). However, adding the adaptation current to the model AN2 made response differences more independent of the mean sound level, as required for an invariant coding of sound direction (Fig. 4B).

We hypothesized that a match of the adaptation time constant of the intrinsic adaptation in AN2 with the peripheral adaptation dynamics was crucial for this improved invariance. When we tested our model with different time constants for central adaptation, the original value obtained by fitting the time course to the current injection data (Fig. 2A) indeed yielded the best classification results (Fig. 4C) and highest information transfer about ILD (Fig. 4D). Thus, we found evidence that the adaptation dynamics in the central direction coding neuron AN2 are matched to the time course of information decay about absolute levels in the periphery and may thus enable level-independent direction discrimination behavior in male grasshoppers.

### Behavioral Tests of Model Predictions

Our experimental data and the simulations indicated that peripheral adaptation to the mean sound level hampered the discrimination of direction by central neurons. The potential solution for this conflict we found was to weigh responses over time according to the availability of information about sound direction, by means of an intrinsic adaptation current in the readout neuron.

**Fig 5:**
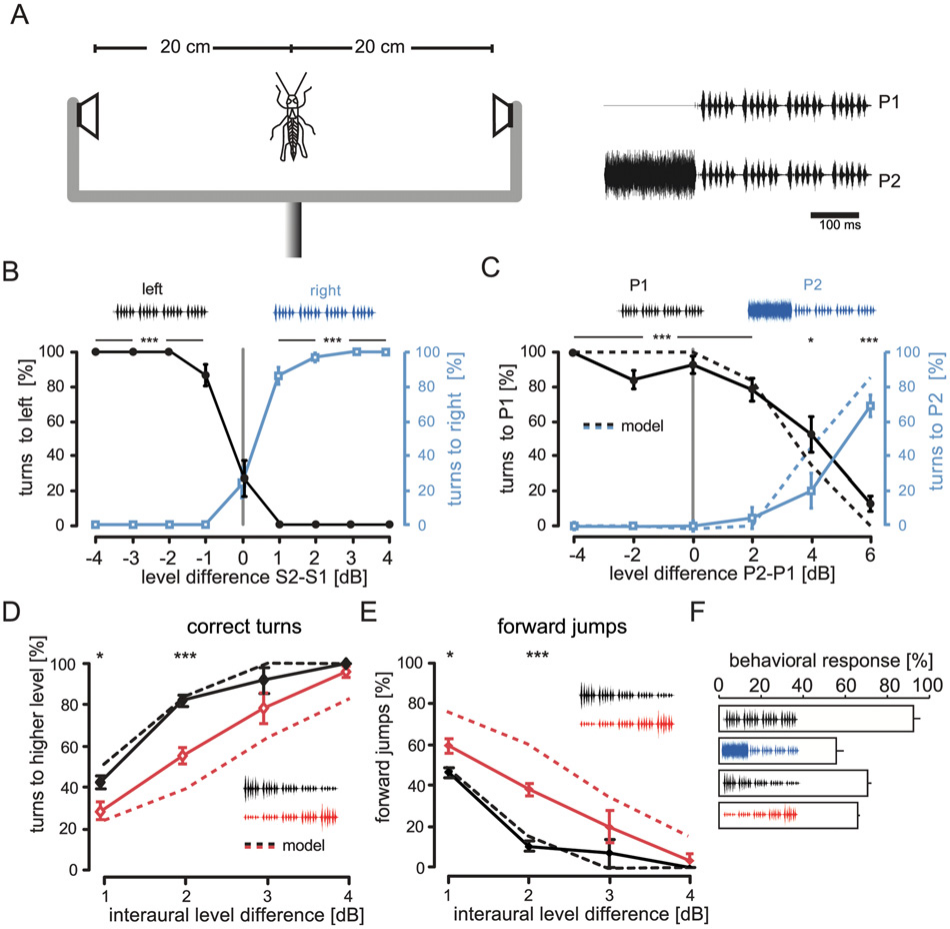
Behavioral tests support model predictions. A: Arrangement of experiments with male grasshopper in the center between the two movable speakers. Right panel shows stimuli that were used in experiments presented in B and C. B: Control experiment with model song (P1 in both speakers). For the analysis, three types of behavioral responses were counted: turns to the left, turns to the right, and forward jumps. The figure contains only turns. Whenever right and left turns are short of 100%, the remaining reactions were jumps. Gray line: equal intensities at both speakers, simulating frontal stimulation. C: Same experiments as in B, but one of the speakers always had a preceding adaptor before the songs were presented (P2). Right and left stimulation was switched randomly during the experiments, plotted in reference to the adaptor stimulus. Dotted line: response of the model. D and E: Behavioral responses to ramped up (black) or down (red) songs. D: Percentage of turns towards louder side (right or left, switched randomly). Asterisks mark significance in a Wilcoxon’s matched pairs signed rank test; * *p* = 0.05, *** *p* = 0.001; E Percentage of forward jumps as a function of inter-aural level difference. Error bars show SEM. F: Overall responsiveness of the males to the four different sets of stimuli presented in B–E: control (B), forward masking (C), ramped down (D and E), and ramped up (D and E). See S3 Data for behavioral data underlying panels A-C and S2 Code for the network model.

From this analysis, specific predictions followed for behavioral experiments in which the ability of grasshoppers to localize sound can be tested. We used the following setup to apply two behavioral paradigms. A male grasshopper is stimulated simultaneously via two loudspeakers (Fig. 5A). Whenever the male produces a calling song, we “respond” with a playback of a female song, with slightly different intensities from the two speakers (ILDs). All stimuli are presented at intensities close to behavioral threshold in order to avoid the effect of sound traveling from the right speaker to the left ear and vice versa [22]. If the male is able to detect which speaker broadcasts the higher sound level, he will reliably turn towards that direction. Very small level differences suffice for a correct localization in the normal stimulus situation [22]. We used short stimuli (340 ms) to establish an open loop situation. The males start to turn only after approximately 500 ms (see [32]). Hence, the stimulus was completed before the behavioral response started, giving time for full analysis of the song. Fig. 5B shows the turning responses to these short songs played at different ILDs (pattern P1 from Fig. 5A). At an ILD of 1 dB the animals already showed a high performance of more than 80% turns to the correct side. However, if males could not resolve the level difference between the speakers, i.e., they perceived the sound as coming from the front, they turned randomly to either side, and in addition, they tended to jump forward ([22,32], see points at 0 dB level difference in Fig. 5B; here approximately 30% turns and 70% forward jumps occurred). At ILDs of 2 dB the number of correct turns was already maximal and indicated that male grasshoppers lateralized the sound source very precisely.

Our model predicted that if an unstructured adapting stimulus was presented mono-laterally just before the onset of the song (Fig. 5A, stimulus P2) the lateralization should be biased towards the other side. In this case, the periphery on the adaptor side had become less sensitive and the relative strength of the inputs from both sides to the central neurons sensitive for direction should be shifted. Therefore, the prediction was that males should turn away from the sound source with the adapting condition P2 and should turn towards the side without adaptor (P1 in Fig. 5A). Although overall responsiveness to the pattern was lower (Fig. 5F), the experimental results nicely exhibited the trend predicted by the model. Even when the song with the preceding adaptation was 4 dB louder, the animals still turned preferentially to the other side (Fig. 5C). Only at a difference of 6 dB, this trend reversed, and the animals correctly turned towards the louder side.

A second prediction of the model was derived from the observation that the central interneuron AN2 responded mainly to the onset of sound patterns (Fig. 2). A stimulus that is slowly ramped up from sub-threshold levels should still be recognizable for males. If the directional response towards a sound source was based only on the onset, however, as the stimulus becomes louder, the periphery is subject to ongoing adaptation, which would strongly reduce directional information (Fig. 3A). Therefore, males were expected to show a reduced lateralization performance for such ramped up stimuli. We chose to play back short grasshopper songs that were either ramped up or down in sound level (Fig. 5D inset). The latter song type should be easier to lateralize for the animals, due to its onset at supra-threshold sound levels. This was indeed the case. At 2 dB ILD the animals reached an 82.7% performance for the downward-modulated songs, whereas the performance was only 55.3% for the upward modulation (Fig. 5D, difference highly significant, *p* < 0.001). The result of this test cannot be explained by the males reacting less to the ramped female songs (Fig. 5F). In addition, when the upward sweep was presented, the animals produced significantly more forward jumps: 37.3% forward jumps in the upward case at 2 dB ILD compared to only 9.8% for the downward modulation (Fig. 5E). This result indicated that with the upward sweep, the tested males could not resolve an ILD of 2 dB and, hence, often classified this stimulus as coming from the front in spite of the 2 dB difference. These results and the results with adapting stimulus (Fig. 5C) further confirmed the prediction of our model and strongly suggested that the animals used the onset of a sound pattern as the most reliable information about the direction of a sound source.

## Discussion

We explored whether a conflict between sound level invariance and direction discrimination exists in the auditory pathway of grasshoppers, and if so, how this conflict could be resolved. Adaptation quickly resulted in extensive level invariance of pattern coding after the first synapse in the periphery (Fig. 1). We employed a computational network model to show that such peripheral adaptation introduced ambiguities for decoding of sound direction based on ILD (Figs. 3 and 4). When we added a strong adaptation current to the central neuron of the model, based on experimental findings, the decoding of direction was restored for a large range of stimuli (Fig. 4). Finally, we tested qualitative predictions of the model in behavioral tests. We confirmed that monaural adaptation biased decoding of ILDs (Fig. 5C) and that directional decoding focused on the onset of the relevant stimulus (Fig. 5D and Fig. 5E). These findings were in accordance with the adaptation properties of both the periphery and the central direction decoding neuron.

### Conflicting Demands of Localization and Pattern Representation on Peripheral Adaptation

**Fig 6:**
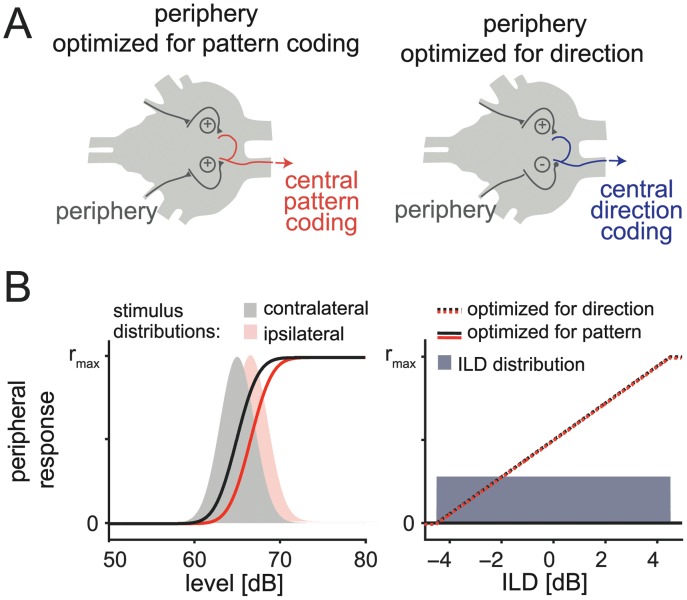
Conflicting demands of localization and pattern representation on peripheral adaptation. A: Schematic drawing of the network architecture. Receptors and local neurons (gray) are subsumed under “periphery” (for more details, see Fig. 1). B: Optimal peripheral response curve for coding of pattern (left) or direction (ILD, right) of a sound. The shaded areas in the left panel represent the amplitude distributions at the ipsi-lateral (gray) and the contralateral (red) side of the animal for lateralized sound. The solid lines represent the optimal response curves of the output of the ipsi- and contra-lateral peripheral network for pattern coding. Different levels or directions demand different peripheral response curves. The shaded area in the right panel represents the distribution of all ILD the animal may experience, restricted by the physical constraints of the ears. Dashed line represents optimal outputs of the periphery for direction coding.

In auditory systems of insects, as well as vertebrates, information from both ears is initially processed independently for each side and then combined, for both pattern- and direction-encoding neurons (see below and Fig. 6A). Adaptation processes generating an intensity invariant representation already in the periphery (receptors and local neurons in the grasshopper) are optimal for pattern encoding independent of sound direction [33]. However, peripheral neurons should not adapt at all for direction encoding in order to preserve information about absolute sound levels at each side. Furthermore, because of ubiquitous noise in neural responses, response-level curves need to be steep to ensure sufficient discrimination of sound levels. This requirement implies narrow response curves because of the limited response range of neuronal firing rates (Fig. 1F). For pattern coding, this is not a problem as long as the response curves are shifted by adaptation to the mean signal intensity (Fig. 6B, left panel). However, for optimal encoding of direction, monotonically increasing response curves covering a broad intensity range that do not adapt would be optimal (Fig. 6B, right panel). These arguments are formalized in S2 Text, and clearly demonstrate the conflict between pattern and direction encoding regarding peripheral adaptation and response curve shapes in the steady-state. The solution to this general problem we present here emphasizes onset transients that still contain directional information before the system is completely adapted.

### Network Layouts and Sensory Adaptation in the Auditory System

Another possible solution to the conflict between AM representation and directional coding addressed here would be to rely on two separate populations of peripheral neurons, one dedicated for coding of ILD and one for pattern representation. As for the receptors, this can be ruled out in grasshoppers. All recorded receptors shifted their response curve via adaptation (Fig. 1G), thereby removing a good part of the centrally available information about direction. For the second neuron in the pathway, the local neuron, we cannot be sure whether another neuron exists that does not add to the receptor adaptation, but based on the current knowledge of the circuitry, this seems unlikely [25]. At this point, the exact wiring pattern of the metathoracic auditory network is only partly known. We know, however, that the local neuron TN1 receives direct input from receptor neurons [34]. TN1 itself acts in an inhibitory manner and is the major candidate for the subtractive input current observed in central direction sensitive neurons [25,35]. In addition, the results of the behavioral experiments with the forward masking adaptor (Fig. 5C) strongly indicated a major contribution of peripheral adaptation on directionality coding.

### The Critical Role of Timescales

As our data and simulations suggest, a separation of pattern and direction encoding in time is a simple, yet powerful, solution to the ambiguity problem introduced by neural adaptation. In particular, a match of peripheral and central time constants is crucial for this process. In addition, this mechanism critically relies on the relation between typical timescales of the signals that need to be processed and the adaptation time constants. From a signal processing point of view, adaptation acts as a high-pass filter and thus does not affect the coding of fast amplitude modulations [30,36]. In the case of the grasshopper song, this applies to the fast amplitude modulations of the stimulus pattern, but also to the sudden availability of directional information at the onset of the stimulus. Multiple timescales of adaptation and temporal coding in the same pathways have been observed in various systems [36–38], even scaling of adaptation dynamics with the frequency of rapid changes of signal [10]. Potentially, some of the variety of timescales found in other systems reflects the dynamics of relevant signals and the need for a temporal separation of information streams, as presented in the current work.

### Behavioral Consequences

Limiting direction discrimination to the onset of a stimulus comes with two major consequences: the inability to trace moving stimuli and the need for a memory trace of the directional information. For grasshoppers, the most relevant sound to be localized are sounds of conspecifics, i.e., of potential mates [31,32]. While the male waits for the female to respond, it sits still, because singing and moving are mutually exclusive—for both actions the legs are used [39]. The memory trace could be placed anywhere between the ascending, directional neuron (e.g., AN2) and the movement apparatus. The directionality could even be stored mechanically in the legs of the grasshoppers [40] while the insect is still evaluating the temporal pattern that eventually triggers the movement.

### Comparison to Avian and Mammalian Auditory System

Because of the larger inter-aural distance, mammals and birds can rely on ILD and inter-aural time differences (ITD) [19]. Neural adaptation and perceptual shifts of perceived direction have been reported for both ITD and ILD coding in mammals [41,42]. In birds, peripheral adaptation has been shown to be detrimental for spike timing precision in the ITD coding pathway [43]. For high-frequency sound, many mammals rely almost entirely on ILD for horizontal localization. The mammalian auditory system relevant to ILD coding shares some of the central features of what has been described here. Peripheral adaptation at similar timescales as in the receptor neurons of the locust has been revealed in recordings from the auditory nerve of guinea pigs [44,45], removing much inter-aural level difference over time [13]. At later stages of the pathway, neurons have been shown to respond invariantly to mean intensity after a short adaptation period [5]. For localization, information from both ears is combined centrally, in neurons of the lateral superior olive (LSO). Similarly to the system presented here, this is done subtractively via excitation and inhibition [46–48]. Behavioral tests using large ILDs in headphone experiments revealed some adaptation to ILDs [41], a test very similar to our monaural forward masking experiments (Fig. 5C). The inputs to central neurons in the LSO display shallow response curve with large dynamic ranges [46], as predicted by our information theoretic calculations (Fig. 6B). Neurons in the LSO and higher brain centers have been shown to code ILDs invariant of mean level [48]. Strikingly, central LSO neurons project back to their inputs via recurrent, presynaptic inhibition [49], which would have a similar effect as the intrinsic, output-driven adaptation described here—both are implementations of a negative feedback loop. Thus, the similarity of the task, to process intensity differences between the two ears while accomplishing intensity variance for pattern recognition at the same time, could have led to different implementations of the same algorithm in such distantly related animal groups as insects and mammals.

## Materials and Methods

### Electrophysiology

All electrophysiology was performed in vivo in *L*. *migratoria*. For receptor recordings (*n* = 7), the auditory nerve was exposed, stabilized with a metal platform, and glass pipets were inserted, until a stable intracellular recording was established. For TN1 recordings (*n* = 10), the metathoracic ganglion was exposed and penetrated with glass pipets. After successful recording, a dye was injected electrophoretically. Post-hoc histology confirmed the identity of the TN1. For a more detailed description of receptor recordings see [23], for details on TN1 recordings [25].

### Stimuli

Receptor neurons were stimulated at their best frequency, as determined on a cell-to-cell basis. All TN1 neurons were stimulated at 5 kHz, which corresponds to their best frequency. Responses at different background levels were obtained using a constant tone that was stepped up or down with a 2 ms ramp to the respective test values [50]. To test for invariant coding of amplitude modulated sounds, we used a randomly amplitude modulated (RAM) stimulus (details: [50]). For each cell, the RAM was played back at approximately 5 dB and 20 dB above threshold of the unadapted responses curve.

### Analysis

To obtain level response-curves, the onset spike frequency was quantified, defined as the average inter-spike interval after each level step [50]. In order to parameterize the shift of the response curves, the curves were parameterized by fitting sigmoid functions to the data. We used the positive part of the hyperbolic tangent:

$$f\left(I\right)=\{\begin{array}{c} f_{\text{max}}\tanh\left(k\left(I-I_{th}\right)\right);I>I_{th}\\ 0;I\leq I_{th} \end{array},$$ (1)

with the slope factor *k* and the position factor *I*_*th*._ For each cell *f*_*max*_ and *k* were held constant among different adaptation conditions.

Responses to the RAM stimuli were expressed by the spike frequency over time for each cell [50]. The response difference was evaluated by subtracting the response to the lower intensity from the response to the higher intensity. Before averaging response differences from all cells, the difference was scaled such that for each cell the maximum difference was one.

### Model: Spiking Neurons

For the circuit model, receptor and local neuron levels were combined into the response of the local neurons [33]. In order to model the response of the periphery, the sound amplitude is transformed by a sigmoidal nonlinearity to the input current of an exponential integrate-and-fire neuron [51]. The model was chosen in order to have a simple model that matched spiking behavior of AN2 given the experimentally observed responses of local neurons and in which an adaptation current could be explicitly entered. A linear integrate-and-fire model failed to reproduce AN2’s response pattern. A pair of directionally sensitive ascending neurons was simulated. The input to each of these is provided by three peripheral neurons from each side and these connections are excitatory from the ipsi-lateral periphery and inhibitory contra-laterally. Inhibitory and excitatory neurons were simulated using the same set of parameters; the only difference between them constitutes their postsynaptic effect. Adaptation at the periphery is implemented by dynamically moving the center of their input-nonlinearity according to the stimulus history weighted exponentially over time. In order to simulate the experimentally observed intrinsic adaptation current in AN2, an output-driven potassium current is added to the peripheral neuron. All parameters were chosen to match experimentally observed response characteristics, spike-train statistics and adaptation dynamics. For a more detailed description of the network layout and model parameters see S1 Text and S1 Table and S2 Table). For the code used for the network model, see S1 Code and S2 Code.

### Data Analysis: Prediction of Direction and Classification

For the time-resolved response to stimuli with amplitude modulations, an artificial grasshopper song was used (the same as in [52]). The songs were presented to the model for 33 different mean intensity levels (48–80 dB in 2 dB steps), at ten different inter-aural intensity levels each (0–9 dB). In order to test the invariance of directionality coding to mean levels, the responses of one ipsi- and one contra-lateral central neuron were compared. In order to do this, the responses of the two neurons *r*_*contra*_ *(t)* and *r*_*ipsi*_ *(t)* were raised to the power of *k* and subtracted from each other: *r* ^*k*^_*ipsi*_ *− r* ^*k*^_*contra*._ Then the average was taken for each mean level and intensity difference. For both model versions, with and without intrinsic adaptation, values of *k* between 2.5 and 3.5 yielded the best results of correct classification of intensity difference, but the adapting model was always better than the non-adapting version. Here, results for *k* = 3 are shown. Each combination of mean level and inter-aural intensity difference was repeated 100 times.

To quantify the classification success of the two model versions, confusion matrices were calculated by taking the temporal average over each response difference, regardless of absolute value. Mean levels between 48–80 dB were used, and each stimulus was repeated 100 times; 3,300 trials were averaged for each intensity difference. Responses of all trials (3,300 times 10 intensity difference = 33,000) were classified to the intensity difference for which the absolute difference to the mean response was smallest. The mean classification result was calculated by evaluating the percentage of trials that were correctly classified for each intensity difference and then averaged over all intensity differences.

### Behavioral Tests

For predictions of behavioral results, the same stimuli were used as in the experiments, each repeated 1,000 times. For each trial the number of spikes on each side (*n*_*contra*_ and *n*_*ipsi*_) were compared and the simulated responses was a turn towards the side with the higher number of spikes. For *n*_*contra*_ = *n*_*ipsi*_, the simulated response was a forward jump.

For the behavioral tests, artificial female grasshoppers songs were constructed [52]. Songs were presented bilaterally from two speakers, each positioned at 20 cm distance from the animals. Pre-tests (not shown) revealed behavioral thresholds around 52 dB. Songs were played back at 54 dB from one speaker and at 54, 55, 56, 57, 58, and 60 dB from the other. In the control experiments, only the artificial songs were played back, pseudo-randomly varying which side was louder. Each condition was tested until we observed at least ten reactions per condition and animal. The reaction consisted either of quick turns towards one side or forward jumps. The control experiment was carried out in eight animals, five of which were also used in the experiments in which the songs were preceded by adaptation noise pulse on one side. The adaptation pulse consisted of 200 ms noise with the same spectrum as the songs, including 5 ms ramps. This pulse was played back at 60 dB, and between the end of the pulse and the beginning of the song patterns we left a 20 ms pause. This experiment was carried out in 11 animals (level differences between −4 and +4 dB) or five animals (+6 dB difference only). For the third experiment, the level of five single syllables constituting the stimulus was either ramped up or down. The level of the loudest syllable (either first or last) was set to 56 dB and the other syllables were reduced by 3 dB each, so that the one played back at the lowest level was at 44 dB and therefore below the previously determined behavioral threshold. These experiments were carried out in eight animals. For further details on the apparatus see Ronacher and Hennig [52].

## Supporting Information

S1 Code ZIP-Archive containing MATLAB code for the model shown in Fig. 2A and Fig. 2B. Contents: **AN2_freq.txt**: Script to run the simulation presented in Fig. 2B and and Fig. 2C. Calls “AN2_networkModel.” **AN2_networkModel.txt**: Function that implements the entire network simulated for Fig. 2. Inputs are the auditory stimulus (amplitude modulation), the inter-aural intensity and a structure holding the initial state of the network fort the simulation. It returns spike trains of the AN2 and the structure holding values for the updated state oft the network. Calls shiftingRec.txt and AN2.txt. **shiftingRec.txt**: Implementation of the adapting receptors neurons as described in S1 Text. Accepts auditory stimulus initial states as inputs and returns voltage traces, spike trains, and new states. **AN2.txt**: This function implements the AN2. It requires excitatory and inhibitory spike trains as inputs and returns the voltage trace, response spike trains, and adaption state variable. It also accepts the value of the adaptation state variable *w* (see S1 Text) and amplitude of adaptation *Δa* (S1 Text) as optional inputs. AN2.txt transforms the input spike trains to an input current and passes it on to adaptEIF.txt. **adaptEIF.txt**: General implementation of an adaptive integrate-and-fire neuron (see S1 Text for description and reference).
(ZIP)

S2 Code ZIP-Archive containing MATLAB code the model used for the simulation results shown in Fig. 3, Fig. 4, and Fig. 5. Contents: **run_model_main.txt**: Script to run the simulation of the network shown in Fig. 3, Fig. 4, and Fig. 5. Calls saveShiftingRec.txt, exampleTraces.txt, saveAN2_varyTimeConstant.txt and tauScript.txt. **saveShiftingRec.txt**: Script that passes artificial songs to the model receptors (shiftingRec.txt) at different levels. The resulting spike trains are saved to disk in order to be used as inputs to AN2.txt. **exampleTraces.txt**: Script that uses the previously saved receptor responses to simulate AN2 responses and plot these. This script was used to generate traces in Fig. 3A and Fig. 3B. Calls AN2.txt. **saveAN2_varyTimeConstant.txt**: Script that uses the previously saved receptor responses to simulate AN2 responses at various levels, ILDs and with varying adaptation time constants. Saves the AN2 responses to disk in order to be analyzed using makeConfusion.txt. Calls testANDiff.txt. **testANdiff.txt**: Simulates the AN2 by loading saved receptors spikes train at a specific combination of intra- and ipsilateral intensities. **AN2.txt**: Implementation oft the AN2, see S1 Code. **adaptEIF.txt**: Adaptive integrate-and-fire neuron model, see S1 Code. **freq4model.txt**: Converts data in spike train format to spike frequency. **makeConfusion.txt**: Function to evaluate classification of the AN2 responses saved in the file provided as input to the function. Plots confusion matrices and returns how many of the ILDs were classified correctly (perCorr1), or within ±1 dB ILD from the presented on (perCorr2) and the information content of the confusion matrix (infoBits). **tauScript.txt**: Script to evaluate model simulation of AN2 with different time constants τ. Loads previously saved model responses, runs makeConfusion.txt, and plots percent correct and information as a function of τ.
(ZIP)

S1 Data Neurophysiological data used to generate panels C–G of Fig. 1. (XLSX)

S2 Data Neurophysiological data used to generate panels A–C of Fig. 2. (XLSX)

S3 Data Behavioral data used to generate panels B–F of Fig. 5. (XLSX)

S1 Fig Network used for numerical simulation. For the numerical simulation, the layered auditory pathway of the locust (Fig. 1B) was reduced to three “local neurons” (LN) that transduce the sound on both sides and directly project onto the central *AN*_*dir*_. The *AN*_*dir*_ receives excitatory input from one side and inhibitory from the other. All local neurons (inhibitory and excitatory) are simulated with the same parameters, the only difference between contra- and ipsi-lateral LNs is the postsynaptic effect in the *AN*_*dir*_.
(EPS)

S2 Fig Example of responses of simulated local neuron. The stimulus used in the simulation is a square pulse of 500 ms duration. The time axis is scaled relative to stimulus onset (0 ms). The uppermost panel shows an example voltage trace of the exponential integrate and fire neuron (EIF) used for the LN with a shifting nonlinearity. The middle panel depicts dot plots of 30 trials of the same stimulus with the same parameters but different noise currents. The lower panel shows the time course of spike frequency, calculated from the 30 trials shown above. Spike frequency was calculated as described in the Materials and Methods section. The time constant of weighting the stimulus history that controls the position of the nonlinearity was 40 ms.
(EPS)

S3 Fig Characterization of adaptation in local neurons. (A): Time course of spike frequency adaptation in simulated local neurons. The responses of a simulated LN to the onset of a pulse stimulus at different intensities are shown. Different colors depict different sound intensities as indicated by the legend. All simulations were run with the same time constant of the leaky integrator driving adaptation (40 ms). The values of τ given in the legend are the results of exponential fits to the time course of the spike frequency for each stimulus level. (B): Shifting of onset response curves as a result of adaptation. The black line marks the unadapted onset response curve of a LN, when silence preceded the test pulse. The colored lines depict the onset response before presentation of the test pulse; the LN was adapted to the background intensity indicated by the dotted lines of the respective color. The spike frequency is calculated as the inverse of the first spike interval.
(EPS)

S4 Fig Responses of simulated local neurons to RAM stimuli. Displayed are responses to the same randomly amplitude-modulated stimulus at different mean levels indicated by the colors of the lines. Spike frequency was quantified by averaging over 30 trials each. The parameters used are the same as in the previous figures. The intensity differences can either be interpreted as the same “object” at different distances or pairwise as inter-aural level differences, stemming from a lateralized sound source.
(EPS)

S1 Table Parameters used for the model of local neurons. (PDF)

S2 Table Parameters used for the model of *AN2*. (PDF)

S1 Text Detailed description of simulation of the network. (PDF)

S2 Text Conflicting demands of localization and pattern representation on peripheral adaptation. (DOCX)

## Abbreviations

AM: amplitude-modulated
ILD: inter-aural level differences
ITD: inter-aural time differences
LSO: lateral superior olive
