# Supplementary material for: A Neural Mechanism for Time-Window Separation Resolves Ambiguity of Adaptive Coding
Source: PLoS Biol. 2015 Mar 11;13(3):e1002096. doi: 10.1371/journal.pbio.1002096 (PMC4356587; doi:10.1371/journal.pbio.1002096)
Supplement: S1 Table — (PDF) [file pbio.1002096.s010.pdf]

Parameter	Meaning	Value
R_M	input resistance	$20\,\mathrm{M\Omega}$
τ_M	membrane time constant	8 ms
E_{leak}	leak reversal potential	-60 mV
V_T	threshold voltage	-50 mV
V_{reset}	reset voltage	-70 mV
Δ_T	slope factor	2 ms
I_{max}	maximal input current	5.5 nA
σ_I	standard deviation of input current	0.35 nA
m	slope of input nonlinearity	$0.15\,\mathrm{dB}^{-1}$
τ_a	time constant of adaptation	40 ms
s_{thr}	absolute threshold of shift	40 dB
k	shift control constant	0.38
