# Supplementary material for: A Neural Mechanism for Time-Window Separation Resolves Ambiguity of Adaptive Coding
Source: PLoS Biol. 2015 Mar 11;13(3):e1002096. doi: 10.1371/journal.pbio.1002096 (PMC4356587; doi:10.1371/journal.pbio.1002096)
Supplement: S2 Table — (PDF) [file pbio.1002096.s011.pdf]

Parameter	Value
τ_{inh} (IPSP decay)	1.5 ms
τ_{exc} (EPSP decay)	4.8 ms
c_{inh} (IPSP amplitude constant)	33.0 mV·ms
c_{exc} (EPSP amplitude constant)	24.9 mV·ms
a (adaptation magnitude)	2 mV
τ_{adapt} (adaptation time constant)	190 ms
