# Supplementary material for: A Neural Mechanism for Time-Window Separation Resolves Ambiguity of Adaptive Coding
Source: PLoS Biol. 2015 Mar 11;13(3):e1002096. doi: 10.1371/journal.pbio.1002096 (PMC4356587; doi:10.1371/journal.pbio.1002096)
Supplement: S1 Text — (PDF) [file pbio.1002096.s012.pdf]

TEXT S1: SIMULATION OF THE NETWORK

Layout of the network used for the simulations

Similar as in the analytical treatment, in the numerical simulation receptor and local neuron levels are combined into the response of the local neurons. For the numerical model three local neurons were used on each side as inputs to the central neurons (Fig. S1) to better match the experimentally observed voltage traces spiking behavior of the central neurons. In order to model the response of the LNs, the sound amplitude is transformed by a sigmoidal nonlinearity to the input current of an exponential integrate-and-fire neuron (EIF). After the transformation, a noise current is added to the input to the EIF. A pair of directionally sensitive ascending neurons (AN_{dir}) was simulated. The input to each of these is provided by three local neurons from each side (Fig. S1) and these connections are either excitatory, if the stem from the ipsilateral LNs or inhibitory, if their origin is contralateral. All LNs were simulated separately and independent noise was added to their inputs. Inhibitory and excitatory neurons were simulated using the same set of parameters; the only difference between them constitutes their postsynaptic effect.

Simulation of local neurons

For the peripheral neurons, a modified version of the exponential integrate-and-fire neuron [Brette and Gerstner, 2005, Fourcaud-Trocmé et al., 2003] was used:

$$\tau_M \frac{dV}{dt} = IR_M + E_{leak} - V + \Delta_T \exp\left(\frac{V - V_T}{\Delta_T}\right). \tag{S1}$$

In eq. S1, τ_M is the membrane time constant, V the membrane potential and t the time. I denotes the input current, R_M the input resistance of the cell, Δ_T a slope factor and V_{thresh} the threshold current. At spike times ($V > 20\,\text{mV}$), $V \to V_{reset}$.

Current input I is generated by passing the acoustic input s through a sigmoidal nonlinearity and adding a constant noise term σ_I:

$$I(s) = \frac{I_{max}}{1 + e^{-m(s - s_{mid})}} \pm \sigma_I. \tag{S2}$$

I_{max} is the maximal current driving the systems and therefore also limits spike frequency, m controls the slope and thus also determines the dynamic range of the response curve and s_{mid} denotes the midpoint of the nonlinearity: at $I(s_{mid}) = 0.5 I_{max}$.

Input-driven adaptation is introduced by the dependence of s_{mid} on the stimulus history:

$$s_{mid}(t) = \int_{-\infty}^{t} s_{rect}(\phi) \exp\left(\frac{(t - \phi)}{\tau_a}\right) d\phi + \frac{\log(k^{-1} - 1)}{m}, \tag{S3}$$

where τ_a is the time constant of adaptation. s_{rect} denotes the stimulus s passed through a halfway rectification with respect to the absolute threshold of the shift s_{thresh}:

$$s_{rect} = \begin{cases} s_{thresh} & \text{if } s \leq s_{thresh} \\ s & \text{else.} \end{cases} \tag{S4}$$

The parameter k appearing in the additional term of eq. S3 lies in the range $0 < k < 1$ and controls which point of the nonlinearity is shifted according to the stimulus history. This means, at $k = 0.5$, the midpoint of the nonlinearity is shifted to the weighted mean of the stimulus history, at lower values of k the adaptation resembles rather a threshold shift. For example, $k = 0.1$ means that the point where the nonlinearity reaches $0.1 I_{max}$ is shifted according to the stimulus history, rather than the midpoint. The values for the parameters used are given in tab. S1.

Response to constant stimuli. Fig. S2 shows the result of the stimulation of a LN with a constant sound pulse played at an intensity above the midpoint of the input-nonlinearity. The LN reacts with a fast onset response that gradually decays as the stimulus presentation persists. This decay is present in all trials. If the mean spike frequency for all trials is plotted over time (Fig. S2, lower panel), an approximately exponential time course with a time constant of the same magnitude as the weighting exponential of the input-adaptation (40 ms in the example of Fig. S2) can be observed.

The time course of adaptation in the LNs approximately matches experimental results [Hildebrandt et al., 2009, Benda, 2002]. In Fig. S3A the spike frequency of an LN in response to the onset of a stimulus pulse played at different sound levels is shown. All values fall well into the range observed in different receptors and candidate LNs (i.e. TN1; Hildebrandt et al., 2009).

The shift of the response curves that result from the adaptation in the simulation are shown in Fig. S3B. The overall shape of the curves is dominated by the input-nonlinearity. If the neuron was adapted to a certain background intensity (dotted lines in Fig. S3)B before testing, the response curves are shifted accordingly. For all following simulations, LNs with the properties described here were used.

The response of the adapting LNs to a randomly amplitude-modulated (RAM) stimulus is displayed in Fig. S4. At the onset of the stimulus, responses diverge strongly, but after about 100 ms, the responses are invariant to the mean stimulus level, because adaptation shifts the input-nonlinearity of the LN accordingly.

Numerical model of the ascending neuron (AN_{dir})

The ascending AN_{dir} is simulated by an EIF type model similar to that of the LNs, but without an additional nonlinearity. For a detailed description, see appendix. It receives excitatory input from three local neurons from one side and from three inhibitory neurons from the other (Fig. S1). Synaptic processes are simulated by transforming input spikes to exponentially decaying positive or negative currents. In addition, an output-driven adaptation current is either added to the AN_{dir}. Each time a spike is elicited in the AN_{dir}, an exponentially decaying, inhibitory current is added to the neuron. If any other parameter is changed, this will explicitly be stated. The amplitude of these steps and their decay rate were chosen in order to match the experimental results [Hildebrandt et al., 2009].

For the voltage course of AN_{dir} the same model as for the local neurons was used and intrinsic adaptation was added to eq. S1:

$$\tau_M \frac{dV}{dt} = IR_M + E_{leak} - V + \Delta_T \exp\left(\frac{V - V_T}{\Delta_T}\right) - w \tag{S5}$$

$$\text{with the adaptation term} \qquad \tau_{adapt}\frac{dw}{dt} = w.$$

τ_{adapt} is the time constant of adaptation. At each spike time ($V > 20\,\mathrm{mV}$), a change of voltage Δ_a due to adaptation is added: $w \to w + \Delta_a$.

The synaptic input to the AN_{dir} I is given by the sum of inhibitory and excitatory synaptic currents I_{inh} and I_{exc}, respectively. For both, each input spike at the time t_{ij} is replaced by an exponentially decaying postsynaptic potential. Subsequently, these potentials are summed over all input neurons i and spikes j. l and l denote the number of input cells (3 in all simulations presented here) and n_l the number of spikes generated by the ith cell:

$$I_{inh,exc}(t) = \frac{c_{inh,exc}}{l\tau_{inh,exc}} \sum_{i=1}^{l} \sum_{j=1}^{n_l} g_{inh,exc}(t - t_{ij}) \tag{S6}$$

$$\text{with} \qquad g_{inh,exc}(x) = \begin{cases} 0 & \text{if } x < 0 \\ exp\left(-\frac{x}{\tau_{inh,exc}}\right) & \text{else,} \end{cases}$$

where $c_{inh,exc}$ is a constant that scales the amplitude of single postsynaptic potentials. All relevant parameters that are not related to the input nonlinearity of the local neurons in tab. S1 also apply to the AN_{dir}. Synaptic and adaptation parameters in eqs. S5 and S6 were fit to resemble the spike frequency time course of the AN2 [Hildebrandt et al., 2009]. The parameters are listed in tab. S2. An example of how the model behaves without intrinsic adaptation can be seen in Fig. 3: in A, the AN_{dir} is not adapting itself. Without intrinsic adaptation, the model fails to reproduces the burst-like behavior of AN2. Instead, spike frequency decays exponentially.

In order to get a more realistic model and to examine the effect of intrinsic adaptation in the AN_{dir}, intrinsic adaptation was fit to match the adaptation behaviour of the AN2 under current stimulation. If acoustic stimulation is applied to the resulting model, its response closely matches that of AN2 (Fig. 3A).

References

J. Benda. *Single Neuron Dynamics — Models Linking Theory and Experiment.* PhD thesis, Humboldt-Universität zu Berlin, 2002.

R. Brette and W. Gerstner. Adaptive exponential integrate-and-fire model as an effective description of neuronal activity. *J Neurophysiol*, 94(5):3637, 2005.

N. Fourcaud-Trocmé, D. Hansel, C. van Vreeswijk, and N. Brunel. How spike generation mechanisms determine the neuronal response to fluctuating inputs. *J Neurosci*, 23 (37):11628–40, 2003.

K. J. Hildebrandt, J. Benda, and R. M. Hennig. The origin of adaptation in the auditory pathway of locusts is specific to cell type and function. *J Neurosci*, 29(8):2626, 2009.
