# Supplementary material for: A Neural Mechanism for Time-Window Separation Resolves Ambiguity of Adaptive Coding
Source: PLoS Biol. 2015 Mar 11;13(3):e1002096. doi: 10.1371/journal.pbio.1002096 (PMC4356587; doi:10.1371/journal.pbio.1002096)
Supplement: S2 Text — (DOCX) [file pbio.1002096.s013.docx]

Text S2:

**Conflicting demands of localization and pattern representation on peripheral adaptation**

In order to assess the consequences of peripheral adaptation for central coding of direction and pattern formally, we calculated the optimal level response curves for both tasks with a stationary model. To this end, we collapsed receptors and local neurons of each side into a single population termed ’periphery‘. The periphery of both sides feeds onto central ascending interneurons. For coding of the pattern, both sides excite a central ascending neuron that sums activity. For directional responses one periphery excites, while the contralateral periphery inhibits a central ascending neuron. We asked how the peripheral response curves *r_per_(x)* should look like in order to maximize information in the central neurons about either direction or the amplitude distribution. The responses of the neurons in the model depend on three variables: the amplitude of the stimulus *x* drawn from the distribution *p_x_*, the mean level of the distribution µ, and the inter-aural level difference Δ*x* the sound receives.

*Pattern coding pathway*

An optimal central response curve of the central pattern coding neuron *r_pat_(x)* should obey three constraints: (1) The response curve should be optimal with respect to the stimulus and therefore proportional to the cumulative probability function, which is the integral of the probability density function *p*_x_ (in the case of constant noise, (Nadal & Parga, 1994)). (2) Responses should be invariant to absolute level µ and inter-aural level difference Δ*x.* (3) *r_pat_* should be the sum of peripheral responses from both sides:

. (1)

The most obvious solution for *r_per_* that also satisfies the invariance criterion are response curves at the periphery that are proportional to the cumulative probability function, but shifted by adaptation to the mean of the stimulus distribution at each side, which is *µ*±Δ*x*. Such adaptation subtracts *µ+∆x* from the argument of the response curves and therefore cancels out the dependence on µ and *∆x*. Thus, adaptation should remove mean level and inter-aural level difference at the level of the periphery independently at each side. Any other solution would be asymmetric and would require directional information not available at the peripheral level.

*Direction coding pathway*

The optimal central response curve of the direction pathway *r_dir_* should (1) be optimal with respect to the distribution of ILDs, (2) be invariant to *x* and µ and (3) be the difference between peripheral responses:

. (2)

The solution we found for the pattern pathway does not satisfy these criteria. If we subtract the adapted peripheral response curves, they would cancel out and no information about the stimulus would be passed on from the periphery. However, a solution equivalent to the pattern pathway but with invariance with respect to (*x+*µ) instead of (*µ*±Δ*x)* is not achievable either. µ cannot be extracted independently without having access to Δ*x,* which is impossible in a purely feed-forward network*.* Adaptation at the periphery does not provide means to adjust the response curves with respect to the mean level of the stimulus in the direction-coding pathway

However, in order to achieve invariance with respect to *x* and µ in the central pattern neuron, we can make use of the subtraction of the peripheries. For any given *∆x* this means that *r_per_(x+µ+∆x) - r_per_(x+µ-∆x) =* *r_dir_(∆x)*. After differentiating this equation with respect to (*x+*µ) and rearranging we get *r_per_‘(x+µ+∆x) = r_per_‘(x+µ-∆x).* The slope of *r_per_* must be constant in the relevant range [x_min_+µ_min_-*∆x_max_, x*_max_+µ_max_+*∆x_max_*]. Thus, to get invariance with respect to (*x+*µ) we need linear, non-adapting response curves in the periphery. This solution is also optimal with respect to the stimulus distribution if we assume uniformly distributed ∆x in the boundaries of the maximal intensity difference between the two ears. In this case, the cumulative probility distribution is a linear relation with a slope of *1/(2∆x_max_)* (Fig. 6B, right panel). Thus, a shallow and linear response curve that covers a wide range of intensities and that does not change with alterations in the signal distribution is preferable over peripheral adaption in the direction coding pathway. In summary, optimal coding for direction and amplitude representation poses contrary demands on the adaptation of the periphery.

Nadal, J. & Parga, N. (1994) Nonlinear neurons in the low-noise limit: a factorial code maximizes information transfer. *Network-Comp. Neural*, **5**, 565–581.
